# A Study of Success Rate of Miniscrew Implants as Temporary Anchorage Devices in Singapore

**DOI:** 10.1155/2015/294670

**Published:** 2015-03-10

**Authors:** Song Yi Lin, Yow Mimi, Chew Ming Tak, Foong Kelvin Weng Chiong, Wong Hung Chew

**Affiliations:** ^1^National Dental Centre Singapore, 5 Second Hospital Avenue, Singapore 168938; ^2^Faculty of Dentistry, National University of Singapore, 11 Lower Kent Ridge Road, Singapore 119083; ^3^Yong Loo Lin School of Medicine, National University of Singapore, 1E Kent Ridge Road, NUHS Tower Block, Level 11, Singapore 119228

## Abstract

*Objective*. To find out the success rate of miniscrew implants in the National Dental Centre of Singapore (NDCS) and the impact of patient-related, location-related, and miniscrew implant-related factors. *Materials and Methods*. Two hundred and eighty-five orthodontic miniscrew implants were examined from NDCS patient records. Eleven variables were analysed to see if there is any association with success. Outcome was measured twice, immediately after surgery prior to orthodontic loading (T1) and 12 months after surgery (T2). The outcome at T2 was assessed 12 months after the miniscrew's insertion date or after its use as a temporary anchorage device has ceased. *Results*. Overall success rate was 94.7% at T1 and 83.3% at T2. Multivariate analysis revealed only the length of miniscrew implant to be significantly associated with success at both T1 (*P* = 0.002) and T2 (*P* = 0.030). Miniscrew implants with lengths of 10–12 mm had the highest success rate (98.0%) compared to other lengths, and this is statistically significant (*P* = 0.035). At T2, lengths of 10–12 mm had significantly (*P* = 0.013) higher success rates (93.5%) compared to 6-7 mm (76.7%) and 8 mm (82.1%) miniscrew implants. *Conclusion*. Multivariate statistical analyses of 11 variables demonstrate that length of miniscrew implant is significant in determining success.

## 1. Introduction

Anchorage has always been one of the most difficult aspects of orthodontic treatment. Traditional methods of anchorage preparation often rely on patients' cooperation and thus may be unpredictable. To ensure attainment of ideal treatment goals, temporary anchorage devices (TADs) are slowly gaining importance with their advantages over the traditional treatment modalities. TADs are devices temporarily fixed to bone for the purpose of enhancing orthodontic anchorage and which are subsequently removed after use. A commonly used TAD would be the miniscrew implant, which is a fixation device placed for anchorage control using mechanical stability without the intention of osseointegration [1]. Miniscrew implants are often chosen among other TADs due to its ease of insertion and removal, relative affordability, and numerous applications in various anatomical locations [2].

In the National Dental Centre of Singapore (NDCS), miniscrew implants were first introduced in the year 2004 but there is currently no available datum on their success rate in NDCS. Success rates seem to vary amongst operators and its use is not widespread due to the purported high dislodgement rate and the need for surgical placement. In the orthodontic literature, there is also no clear information on whether patient-related, location-related, or miniscrew implant-related factors influence the success of miniscrews in NDCS. Meta-analyses [3, 4] conducted have shown that a myriad of factors seem to affect their failure rates, but most variables still need additional evidence to support any possible associations. This is due to the extensive types and brands of miniscrew implants used and the heterogeneity of the included studies which may affect the success rates reported.

Thus, the aim of this retrospective study is to find out the success rate of miniscrew implants in NDCS pertaining to our local population, and whether they are a reliable form of TAD. Secondary objectives of this research will include finding out if patient-related factors, location-related factors, and miniscrew implant-related factors have any impact on success rates.

## 2. Materials and Methods

Records of patients who received miniscrew implants as part of their orthodontic treatment plan during the period of January 2010 to June 2012 were retrospectively examined. This amounted to 136 patients with a total of 285 miniscrew implants. Details of these patients were obtained from the surgical logbooks maintained in the Day Surgery Department in NDCS.

Patients with the following data on the electronic dental records of NDCS were included:

- comprehensive demographic information including dental and skeletal relationships,
- dates of miniscrew placement, miniscrew loading, and miniscrew removal or dislodgement,
- type, length, and diameter of miniscrew,
- location of the miniscrew.

Smokers and patients with systemic medical conditions or those on long-term medications were excluded.

To see if there is any association with clinical success of miniscrew implants, 11 variables were collected for analysis. The 11 variables were divided into 3 categories: patient-related, miniscrew implant location-related, or miniscrew implant design-related factors as shown in Table 1.

Patient-related factors include the age and gender of the patient, the dental malocclusion according to the British Standards Institute incisor classification, and the skeletal (sagittal and vertical) relationship based on the orthodontist's clinical diagnosis and documentation.

Location-related factors of the miniscrew include the side of placement (right, left, or at the midline) and the jaw involved (maxilla or mandible). The miniscrew position in the oral cavity (anterior region, posterior region, retromolar, palate) was also examined. The anterior region refers to the labial dentoalveolus mesial to the canines. The posterior region refers to the buccal dentoalveolus distal to the canines, the tuberosity area and the infrazygomatic crest area.

Miniscrew implant-related factors include the type (VectorTAS or AbsoAnchor) of miniscrew, its length (6 mm, 7 mm, 8 mm, 10 mm, 12 mm), and its diameter (1.3 mm, 1.4 mm, 2.0 mm).

The miniscrew implant placement surgery was done by randomly assigned periodontists or oral and maxillofacial surgeons working in NDCS. Full consent was taken before the surgical procedure. The patients were also instructed on standard postoperative care instructions after the surgery. They were told to brush the surgical site gently to maintain good oral hygiene and a bottle of 0.2% chlorhexidine mouth rinse was prescribed to be used twice daily for a week.

This study examines early and late successes of the miniscrews at 2 time points: on the day of orthodontic loading and 12 months after insertion of the miniscrew implant. The outcome examined at the first time point (T1) will be the miniscrew implant's initial stability, prior to orthodontic loading. Success of the miniscrew implant at that juncture is defined by absence of infection of the surrounding soft tissues or any reason warranting its immediate removal or replacement prior to loading. Failure of the miniscrew implant is defined as dislodgement of the miniscrew implant prior to loading or a miniscrew that have become excessively mobile such that orthodontic anchorage objectives cannot be met. Likewise, if the miniscrew implant has caused irreversible biological damage to adjacent structures as recorded by the clinician and was thus unusable, it was also considered a failure.

The outcome at the second time point (T2) was assessed 12 months after the miniscrew's insertion date or after its use as skeletal anchorage has ceased, whichever came first. Success of the miniscrew implant at this juncture is defined by no dislodgement from the date of initial loading to the 12-month mark after the date of insertion or when intentional removal is carried out prior to the 12-month mark. It will mean that the miniscrew has sustained orthodontic loading forces throughout that time period and has served its skeletal anchorage function. Similarly, failure of the miniscrew will be defined as dislodgement from the surgical site after orthodontic loading, any time before the 12-month period.

The research protocol was approved by the SingHealth Institutional Review Board with CIRB reference 2012/1057/D.

Descriptive statistics were initially performed to calculate the overall success rate of the miniscrew implants, as well as their specific success rates with regard to the 11 variables studied. Multiple miniscrew implants in a patient were assumed to be independent entities. Logistic regression was used to evaluate factors associated with the success of miniscrew implant. The datum was analyzed using SAS version 9.2. Statistical significance was set at 5%. For any pairwise comparisons in the univariate analyses, the Bonferroni technique was applied. The Hosmer-Lemeshow test was used to test for goodness of fit for the logistic regression model and results showed a good fit (at T1, *P* = 0.70; at T2, *P* = 0.11).

## 3. Results

The overall success rate was 94.7% at T1 (95% CI 92.1%–97.3%) and 83.3% at T2 (95% CI 78.7%–87.9%). The detailed information on success rates at T1 and T2 is shown in Tables 2 and 3.

**Figure 1:**
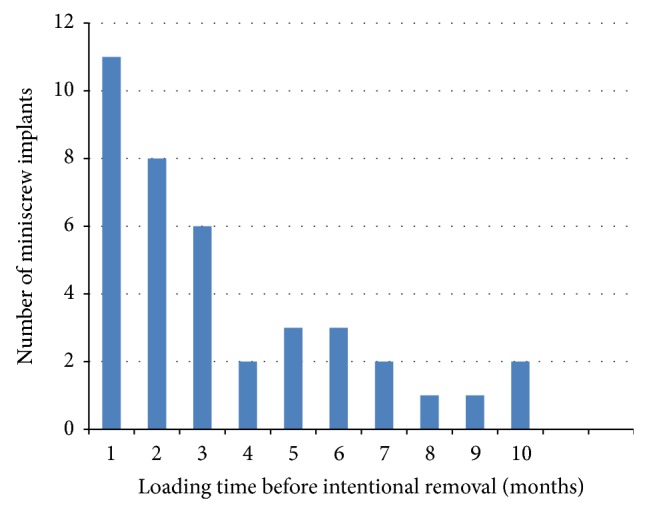
Loading time of successful miniscrew implants removed intentionally.

**Figure 2:**
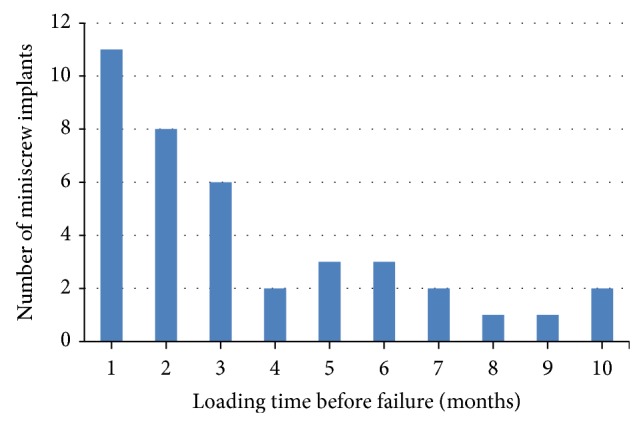
Loading time of failed miniscrew implants.

Out of the 214 successful miniscrew implants at T2, 37 of them were removed intentionally prior to the 12-month mark. These 37 miniscrews had a successful loading duration ranging from 2 to 12 months, and this is presented in Figure 1. Mean loading time for failed miniscrews at T2 was 3.5 months, ranging from 1 to 10 months and this is shown in Figure 2.

### 3.1. Success Rate at T1

In the univariate analyses, length of miniscrew was significantly associated with success at T1 (*P* = 0.001). In the multivariate analysis of success rate at T1, only length of miniscrew implant was still found to be significantly associated (*P* = 0.002) with miniscrew implant success after being adjusted for age, gender, vertical skeletal malocclusion, recipient jaw, and type of miniscrew implant. Due to multicollinearity, some variables in the univariate analyses were not included in the multivariate analysis.

### 3.2. Success Rate at T2

In the univariate analyses, sagittal skeletal malocclusion (*P* = 0.025) and vertical skeletal malocclusion (*P* = 0.028) were significantly associated with miniscrew implant success at T2. Multivariate analysis of the success of miniscrew implants at T2 found vertical skeletal malocclusion (*P* = 0.043) and length of miniscrew (*P* = 0.030) to be significantly associated with success rate.

### 3.3. Patient-Related Factors

Of the patient-related factors, there were no statistically significant differences between the variables at T1. But using univariate analyses at T2, there were associations between sagittal skeletal malocclusion and miniscrew implant success and also between vertical skeletal malocclusion and miniscrew implant success. Miniscrew implants placed in patients with class III malocclusion had a lower chance of success compared with those placed in patients with class I malocclusion (*P* = 0.01, OR = 0.26, 95% CI 0.08–0.79). Miniscrew implants in average angle patients had a higher chance of success compared with those placed in high angle patients (*P* = 0.025, OR = 3.18, 95% CI 1.13–8.98). After adjusting for age, gender, sagittal skeletal malocclusion, dental malocclusion, recipient jaw, type of miniscrew implant, and length of miniscrew, vertical skeletal malocclusion was still found to be significantly associated (*P* = 0.043) with miniscrew implant success. Miniscrew implants in average angle patients had a higher chance of success compared with those placed in high mandibular plane angle patients (*P* = 0.013, OR = 4.22, 95% CI 1.35–13.16).

### 3.4. Location-Related Factors

None of the location-related factors was significantly associated with success at T1 and T2. Although at T1, for side of placement, there seem to be higher success rates for miniscrew implants placed in the midline (100%) compared to the left (94.9%) or ride side (94.5%). This was also reflected at T2; midline miniscrew implants had a 100% success rate compared to the left (85.4%) or right (80.8%). For recipient jaw, success rate of miniscrew implants in the mandible is higher at both T1 and T2 compared to the maxilla. But this is also not significant. Similarly, the different sites of placement had no significant difference in success rates, although the retromolar area showed the highest success at T1 (100%) and T2 (94.4%).

### 3.5. Miniscrew Implant-Related Factors

Of the miniscrew implant-related factors, only length of miniscrew implant was significantly associated with success in the multivariate analyses at T1 (*P* = 0.002) and at T2 (*P* = 0.030). Those with length 8 mm and 10–12 mm had a higher chance of success at T1 compared to those with length 6-7 mm, respectively (8 mm: OR = 11.88, 95% CI 2.73–51.71, *P* = 0.001; 10–12 mm: OR = 10.50, 95% CI 1.18–93.51, *P* = 0.035). At T2, those with length 10–12 mm were found to have a higher chance of success compared with those with 6-7 mm (OR = 17.95, 95% CI 1.83–176.01, *P* = 0.013). Type of miniscrew implant and diameter had no significant association with miniscrew implant success.

## 4. Discussion

The success rate of miniscrew implants in our study was 94.7% at T1 and 83.3% at T2. Success rate at T1 is comparable to the success rate by Lim et al. [5] who reported a 93.1% success rate when they assessed initial stability of the miniscrews 1 week after placement. Similarly, success rate at T2 is comparable to the rates in other retrospective studies of Asian patients, (83.8%–89.9%) [6–9]. This is in spite of the various miniscrew implant systems used, the varying operators and surgical techniques, and diverse management protocols reported by the different centres.

The mean loading time for failed miniscrews in this study was 3.5 months, ranging from 1 to 10 months. Most of the failures (30 out of 39) occurred within the first 5 months after loading. This is in accord with the findings [10] which estimated that the highest failure rate occurred during the first 50–150 days following loading.

Although a success rate of 83.3% is reasonable, there is still a 1 in 5 chance of failure using miniscrew implants for orthodontic anchorage. Schätzle et al. [11] demonstrated that palatal implants and miniplates showed a better survival rate compared to miniscrews. It will be interesting to find out how the success rate of other skeletal anchorage systems is compared against miniscrew implants in NDCS, and whether they can provide an improved and significantly more reliable form of TAD for orthodontic use. This will be elucidated in a future study.

### 4.1. Limitations of Study

Due to the retrospective nature of this study, datum was sometimes lacking and not every variable mentioned in the literature was investigated and confounding factors may be present.

The miniscrew implant placement surgery was done by randomly assigned periodontists or oral and maxillofacial surgeons working in NDCS. Other than standard postoperative care instructions given to the patient, surgical techniques and surgical experience of the clinician may vary and affect the results of our study. Operator's surgical experience in miniscrew placement has been investigated in the literature [5], but this variable was excluded as we felt it was difficult to classify clinicians into groups according to years of experience or number of miniscrews inserted. This is because some clinicians do not work full time in NDCS, and it will be inaccurate to place a clinician in the “inexperienced” group who may have had prior experience in other centres before operating in NDCS.

Unlike a study in laboratory settings, insertion torque, loading forces, and direction of insertion were not recorded to numerical precision on a routine clinical basis. Thus, no data on the above variables could be obtained from the patient charts and treatment note records. Also, it is clinically hard to record accurately a constant magnitude of force due to the rapid force level decay of orthodontic elastomeric chains, which are most commonly used in NDCS for orthodontic loading.

The effect of delayed, early, or immediate loading on success rates was also not investigated as the individual patient's orthodontic appointment varies after insertion of the miniscrew implant and there are no standard loading protocols followed by the orthodontists.

Types of tooth movement involved were investigated by other studies [12] on success rates but this was not investigated as miniscrew implants are sometimes used for a combination of movements (e.g., both intrusion and distalization), thus making it difficult for any meaningful comparison of success rates to be made between any particular tooth movement.

### 4.2. Patient-Related Factors

Using univariate analysis at T2, sagittal skeletal malocclusion was associated with success rate. Miniscrew implants placed in patients with class III malocclusion had lower success compared with class I malocclusion. However, according to studies by Antoszewska et al. [12] and Miyawaki et al. [6], among groups with different skeletal patterns, there are no significant differences in success. There is no obvious physiological reason why dentoalveolar abnormality or malocclusion type should affect success rate. Hence, our initial finding may just be due to chance.

Using a multivariate analysis of success at T2, vertical skeletal malocclusion was significantly associated with success rate of miniscrew implants. This was agreed upon by Antoszewska et al. [12] who found that, out of all the patient-related factors, only the vertical dimension seemed to play a role in determining success rates. Our results showed that average mandibular plane angle patients had a significantly higher success rate compared to high mandibular plane angle patients. This corresponds with the study by Miyawaki et al. [6] who reported that the average mandibular plane angle group had significantly higher success rates compared to the high mandibular plane angle group. It was found that density of cortical bone was higher in subjects with small Frankfort-mandibular plane angles and gonial angles [13]. Accordingly, high mandibular angle patients may have less dense cortical bone and this might affect success rates of miniscrew implants placed. This is supported by results of a meta-analysis [14] which showed a positive association between the primary stability of miniscrew implants and cortical bone thickness of the surgical site.

### 4.3. Miniscrew Location-Related Factors

None of the location-related factors was significantly associated with success at both T1 and T2. For side of placement, at both T1 and T2, success rates for miniscrew implants placed in the midline were the highest, followed by the left then the right side but this did not reach statistical significance. Park et al. [15] and Wu et al. [9] reported that the left side had significantly higher success rates than the right side. In this study, placement of miniscrew implants on the left side does has a slightly higher success rate compared to the right side at both T1 and T2. This may be because most surgeons are right-handed, making it easier to insert miniscrews on the patient's left side. Also, there may be better hygiene maintenance on the left side in right-handed patients, who are most prevalent in the population. Miniscrews located in the midline had the highest success rate in our study and these were all located in the palate. This is similar to the results of a study by Lim et al. [5] which showed a 100% success rate in the mid-palatal area. Reasons for a high success rate in the mid-palatal region might be due to the abundance of compact bone and thin gingival tissue in the area, optimizing miniscrew implant insertion. The success rate of miniscrew implants in the mandible is higher compared to the maxilla at both T1 and T2 but this is not significant. This concurred with results from studies by Miyawaki et al. [6] and Lim et al. [5] who found no statistically significant association with success rates in the maxilla or mandible. The slightly higher success rate in the mandible may be attributed to thicker cortical bone in the mandible which is ideal for miniscrew implant stability [16].

The different sites of placement had no significant difference in success rates in our study, and this supports the results by Chen et al. [17] who showed that placement site (maxilla or mandible, left or right side, anterior or posterior) presented no statistically significant association with success rates. This is in contrast to the study by Tseng et al. [18] who found that the only statistically significant factor affecting miniscrew success rates was location. Success rates were the highest in the anterior tooth-bearing region of the maxilla, followed by the posterior tooth-bearing region of the maxilla, and success declines correspondingly in the anterior dentoalveolus of the mandible, posterior dentoalveolus of the mandible, and lastly the ramus. Chen et al. [7] also observed that the differences in success rates were significant in the different sites: success rate was best in maxillary anterior dentoalveolus followed by maxillary posterior dentoalveolus and then lastly in the mandibular posterior dentoalveolus.

In this study, the success rates of miniscrews were compared at the anterior or posterior dentoalveolus separately from those inserted in the maxillary or the mandibular basal bone. Since both maxillary and mandibular anterior miniscrews are grouped into one general category and vice versa for the posterior miniscrews, this may have decreased the statistical significance of the results.

### 4.4. Miniscrew Implant-Related Factors

Of the miniscrew implant-related factors, only length of miniscrew implant was significantly associated with success at both T1 and T2. Lengths of 10–12 mm had the highest success rate, followed by 8 mm and then the 6-7 mm lengths. This is probably due to the fact that longer miniscrews have the highest contact surface area for mechanical retention. This is in accord with the findings of Chen et al. [7] who found that length of microimplant is a significant risk factor. Success rate for the longer microimplant (8 mm) used in their study was significantly higher than the shorter microimplant (6 mm). Similarly, Tseng et al. [18] found that as success rate increases with length, it was the highest for miniscrews with lengths 12 mm and 14 mm.

Diameter of miniscrew implant had no statistically significant association with success in our study though it shows increasing success with increasing diameters.

Type of miniscrew implant showed no significant association with success although higher success rates were reported for the VectorTAS miniscrews compared to Absoanchor microimplant at both T1 and T2. This could be due to the larger diameter of VectorTAS miniscrews used in NDCS. In NDCS, the more popular AbsoAnchor microimplants used are the small head (SH1312) series, which has a diameter of 1.3 mm only and the lengths used in our study sample range from 6 to 10 mm, depending on the site of placement. In contrast, the VectorTAS miniscrews used in this study sample have a diameter of at least 1.4 mm or 2.0 mm, and lengths that range from 6–12 mm. Due to the larger diameter and longer length of the VectorTAS miniscrews, success rates may be similarly increased. However, since there are no prior studies evaluating the success rates of the two types of miniscrew implants, no comparisons can be made.

## 5. Conclusion

The overall success rate is 83.3% after 12 months. Patient-related factors like vertical skeletal malocclusion were found to influence success: average mandibular plane angle patients have a higher chance of success compared to high mandibular angle patients probably due to the less dense cortical bone of the latter. Miniscrew implant location-related factors have no significant effect on success but careful site selection must still be done to avoid encroaching on vital structures and to optimize orthodontic mechanics. Of the miniscrew implant-related factors, only length of miniscrew implant was significantly correlated with success. Thus, as long as surrounding anatomy permits, a longer miniscrew implant for better mechanical retention is recommended for higher success rate.

## Figures and Tables

**Table 1:** Clinical variables examined.

Categories	Variables	
Patient-related	Age	<20/≥20 years old
	Gender	Male/Female
	Skeletal malocclusion (sagittal)	Class I/II/III
	Skeletal malocclusion (vertical)	High/average/low angle
	Dental malocclusion	Class I/II/III

Location-related	Side	Right/left/midline
	Jaw	Maxilla/mandible
	Position	(Anterior region/posterior region/retromolar/palate)

Miniscrew-related	Type	AbsoAnchor/VectorTAS
	Length	6-7/8/10–12 mm
	Diameter	1.3/1.4/2.0 mm

**Table 2:** Success rate of miniscrew implants at T1.

	Success rate at T1 (%)	Number of 1st outcome successes/Total number	Unadjusted odds ratio (95% CI)	Unadjusted *P* value	Adjusted odds ratio (95% CI)	Adjusted *P* value
Overall success	94.7	270/285				
Age at surgery				0.877		0.251
<20 years	94.6	193/204	0.91 (0.28 to 2.95)		2.31 (0.55 to 9.62)	
≥20 years	95.1	77/81	1		1	
Gender				0.502		0.288
Female	93.8	120/128	1		2.01 (0.55 to 7.31)	
Male	95.5	150/157	1.43 (0.50 to 4.05)		1	
Sagittal skeletal malocclusion				0.421		
Class I	92.6	87/94	1			
Class II	94.7	126/133	1.45 (0.43 to 4.82)	1		
Class III	100	36/36	6.26 (0.22 to 177.92)	0.523		
Vertical skeletal malocclusion				0.918		0.524
High angle	93.8	106/113	1		1	
Average	95.1	97/102	1.28 (0.332 to 4.940)	1	2.12 (0.55 to 8.18)	0.276
Low angle	94.1	16/17	1.06 (0.09 to 12.50)	1	2.38 (0.19 to 29.38)	0.499
Dental malocclusion				0.361		
Class I	96.5	55/57	1			
Class II Div. I	93.4	155/166	0.61 (0.11 to 3.42)	1		
Class III	100	41/41	3.74 (0.09 to 164.50)	0.554		
Class II Div. 2	87.5	7/8	0.23 (0.01 to 3.55)	0.316		
Side of placement				0.928		
Left	94.9	129/136	1.07 (0.34 to 3.41)	1		
Midline	100	4/4	0.56 (0.01 to 25.32)	1		
Right	94.5	137/145	1			
Recipient jaw				0.290		0.286
Maxilla	94.1	222/236	1		1	
Mandible	98.0	48/49	3.03 (0.39 to 23.58)		3.40 (0.36 to 32.15)	
Site of placement				0.647		
Anterior region	93.3	14/15	0.36 (0.03 to 4.96)	1		
Posterior region	93.4	169/181	0.50 (0.09 to 2.76)	1		
Retromolar	100	20/20	1.52 (0.03 to 70.74)	1		
Palate	97.1	67/69	1			
Miniscrew implant type				0.606		0.887
AbsoAnchor	92.6	25/27	1		1	
Vector TAS	94.9	244/257	1.50 (0.32 to 7.04)		1.14 (0.19 to 6.85)	
Miniscrew length				0.001^*^		0.002^*^
6-7 mm	82.7	43/52	1		1	
8 mm	97.3	177/182	7.41 (2.01 to 27.38)	0.001^*^	11.88 (2.73 to 51.71)	0.001^*^
10–12 mm	98.0	50/51	10.47 (0.94 to 116.31)	0.058	10.50 (1.18 to 93.51)	0.035
Miniscrew diameter				0.601		
1.3 mm	92.9	26/28	0.30 (0.02 to 4.86)	0.658		
1.4 mm	94.3	200/212	0.38 (0.04 to 4.02)	0.714		
2.0 mm	97.8	44/45	1			

^*^ *P* ≤ 0.05.

**Table 3:** Success rate of miniscrew implants at T2.

	Success rate at T2 (%)	Number of 2nd outcome successes/Total number	Unadjusted odds ratio (95% CI)	Unadjusted *P* value	Adjusted odds ratio (95% CI)	Adjusted *P* value
Overall success	83.3	214/257				
Age at surgery				0.082		0.270
<20 years	80.7	151/187	0.47 (0.20 to 1.10)		0.49 (0.14 to 1.73)	
≥20 years	90.0	63/70	1		1	
Gender				0.482		0.109
Female	81.4	92/113	1		1	
Male	84.7	122/144	1.27 (0.66 to 2.44)		2.12 (0.85 to 5.31)	
Sagittal skeletal malocclusion				0.025^*^		0.487
Class I	89.5	77/86	1		1	
Class II	83.5	101/121	0.59 (0.23 to 1.54)	0.438	0.88 (0.25 to 3.19)	0.852
Class III	68.6	24/35	0.26 (0.08 to 0.79)	0.014^*^	0.25 (0.02 to 2.56)	0.240
Vertical skeletal malocclusion				0.028^*^		0.043^*^
High angle	79.6	82/103	1		1	
Average	92.6	87/94	3.18 (1.13 to 8.98)	0.025^*^	4.22 (1.35 to 13.16)	0.013^*^
Low angle	75.0	12/16	0.77 (0.19 to 3.13)	1	2.20 (0.36 to 13.33)	0.391
Dental malocclusion				0.260		0.770
Class I	88.9	48/54	1		1	
Class II Div. I	84.0	126/150	0.69 (0.22 to 2.16)	1	0.81 (0.18 to 3.70)	0.787
Class III	75.0	30/40	0.39 (0.03 to 2.66)	1	1.25 (0.09 to 17.71)	0.339
Class II Div. 2	71.4	5/7	0.30 (0.10 to 1.47)	1	0.22 (0.01 to 5.02)	0.868
Side of placement				0.590		
Left	85.4	105/123	1.38 (0.65 to 2.93)	1		
Midline	100	4/4	2.18 (0.05 to 94.33)	1		
Right	80.8	105/130	1			
Recipient jaw				0.081		0.065
Maxilla	81.4	175/215	1		1	
Mandible	92.9	39/42	2.97 (0.87 to 10.10)		9.59 (0.87 to 105.86)	
Site of placement				0.404		
Anterior region	92.9	13/14	2.45 (0.18 to 33.59)	1		
Posterior region	80.9	131/162	0.80 (0.31 to 2.07)	1		
Retromolar	94.4	17/18	3.21 (0.24 to 43.06)	0.848		
Palate	84.1	53/63	1			
Miniscrew implant type				0.508		0.769
AbsoAnchor	78.3	18/23	1		1	
Vector TAS	83.7	195/233	1.43 (0.50 to 4.07)		1.22 (0.33 to 4.53)	
Miniscrew length				0.108		0.030^*^
6-7 mm	76.7	33/43	1		1	
8 mm	82.1	138/168	1.39 (0.55 to 3.52)	0.843	2.91 (0.93 to 9.13)	0.067
10–12 mm	93.5	43/46	4.34 (0.91 to 20.75)	0.071	17.95 (1.83 to 176.01)	0.013^*^
Miniscrew diameter				0.122		
1.3 mm	79.2	19/24	0.20 (0.03 to 1.41)	0.128		
1.4 mm	81.2	156/192	0.22 (0.04 to 1.19)	0.089		
2.0 mm	95.1	39/41	1			

^*^ *P* ≤ 0.05.
